# Book Reviews

**Published:** 1900-08

**Authors:** 


### BOOK REVIEWS, PAMPHLETS, EXCHANGES.

### BOOK REVIEWS.

[Contributions solicited for review.]

A TEXT-BOOK OF PRACTICAL THERAPEUTICS: With Special Reference to the Application of Remedial Measures to Disease and their Employment upon a Rational Basis. By Hobart Amory Hare, M.D., Professor of Therapeutics and Materia Medica in the Jefferson Medical College of Philadelphia. With special chapters by Drs. G. D. deSchweinitz, Edward Martin and Barton C. Hirst. New (8th) edition. In one octavo volume of 796 pages, with 37 engravings and 3 colored plates. Cloth $4; leather $5, net. Lea Brothers & Co., Philadelphia and New York.

The eighth edition of this popular work is just issued. It has been revised and additions made bringing it up to date. The value of Hare's Therapeutics is attested by the popularity it has enjoyed for years. It has a position all its own in medical literature, and is one of the most reliable publications of its character.

TIRARD'S MEDICAL TREATMENT. A Text-book of Medical Treatment of Diseases and Symptoms, for the use of Students and Practitioners of Medicine. By Nestor Tirard, M.D., F.R. C.P., Professor of Principles and Practice of Medicine, King's College, London. Adapted to the U. S. Pharmacopœia, by E. Quin Thornton, M.D., of Jefferson Medical College, Philadelphia. In one octavo volume of 624 pages. Just ready. Cloth $4, net. Lea Brothers & Co., Philadelphia and New York.

Books of this character are peculiarly needed, because there is a tendency to neglect therapeutics for laboratory work. For the active practitioner this work is especially commendable, as it gives in detail the most approved treatment of each disease, including necessary particulars about the disease. Its formulæ have been adapted to the U. S. Pharmacopœia. It will be found highly useful and is sure to prove popular.

LECTURES UPON THE PRINCIPLES OF SURGERY. Delivered at the University of Michigan by Chas. B. Nancrede, A.M., M.D., LL.D., Professor of Surgery and of Clinical Surgery; Emeritus Professor of General and Orthopedic Surgery, Philadelphia Polyclinic, etc., etc. With an appendix containing a résumé of the principal views held concerning inflammation, by Wm. A. Spitzley, A.B., M.D., Senior Assistant in Surgery, University of Michigan. Illustrated. Price $2.50, net. Philadelphia. W. B. Saunders, 925 Walnut street. 1899.

This book is made up of the substance of thirty-six lectures delivered by the author to his class at the University of Michigan, and covers very carefully and concisely the ground plan of surgery. The book can be commended as a text-book and as meriting perusal by the graduate.

THE ESSENTIALS OF HEMATOLOGY. A Practical Guide to the Clinical Examination of the Blood for Diagnostic Purposes. Illustrated. Published by the Palisade Manufacturing Company, Yonkers, N. Y.

This little brochure is a guide to the clinical examination of the blood and is illustrated with a number of cuts. The little work will prove useful. It has the characteristic elegance seen in all the productions of this house.

